# Toward a Regulatory Qualification of Real-World Mobility Performance Biomarkers in Parkinson’s Patients Using Digital Mobility Outcomes

**DOI:** 10.3390/s20205920

**Published:** 2020-10-20

**Authors:** Marco Viceconti, Sabina Hernandez Penna, Wilhelmus Dartee, Claudia Mazzà, Brian Caulfield, Clemens Becker, Walter Maetzler, Judith Garcia-Aymerich, Giorgio Davico, Lynn Rochester

**Affiliations:** 1 Department of Industrial Engineering, Alma Mater Studiorum-University of Bologna, 40136 Bologna, Italy; giorgio.davico@unibo.it; 2 Medical Technology Lab, IRCCS Istituto Ortopedico Rizzoli, 40136 Bologna, Italy; 3 Regulatory Affairs, Novartis Pharma AG, 4001 Basel, Switzerland; sabina.hernandez_penna@novartis.com (S.H.P.); wim.dartee@novartis.com (W.D.); 4 Department of Mechanical Engineering and Insigneo Institute for in silico Medicine, University of Sheffield, Sheffield S10 2TN, UK; c.mazza@sheffield.ac.uk; 5 SFI Insight Centre for Data Analytics, University College Dublin, 716 7777 Dublin, Ireland; b.caulfield@ucd.ie; 6 Department of Geriatric Medicine, Robert Bosch Gesellschaft für Medizinische Forschung mbH, 70376 Stuttgart, Germany; clemens.becker@rbk.de; 7 Department of Neurology, Christian-Albrechts-Universität zu Kiel, 24118 Kiel, Germany; w.maetzler@neurologie.uni-kiel.de; 8 Barcelona Institute for Global Health, 08003 Barcelona, Spain; judith.garcia@isglobal.org; 9 Departament de Ciències Experimentals i de la Salut, Universitat Pompeu Fabra (UPF), 08002 Barcelona, Spain; 10 CIBER Epidemiología y Salud Pública (CIBERESP), 28029 Madrid, Spain; 11 Institute of Translational and Clinical Research, Newcastle University, Newcastle NE1 7RU, UK; lynn.rochester@newcastle.ac.uk

**Keywords:** inertial measurement unit, mobility performance, regulatory science

## Abstract

Wearable inertial sensors can be used to monitor mobility in real-world settings over extended periods. Although these technologies are widely used in human movement research, they have not yet been qualified by drug regulatory agencies for their use in regulatory drug trials. This is because the first generation of these sensors was unreliable when used on slow-walking subjects. However, intense research in this area is now offering a new generation of algorithms to quantify Digital Mobility Outcomes so accurate they may be considered as biomarkers in regulatory drug trials. This perspective paper summarises the work in the Mobilise-D consortium around the regulatory qualification of the use of wearable sensors to quantify real-world mobility performance in patients affected by Parkinson’s Disease. The paper describes the qualification strategy and both the technical and clinical validation plans, which have recently received highly supportive qualification advice from the European Medicines Agency. The scope is to provide detailed guidance for the preparation of similar qualification submissions to broaden the use of real-world mobility assessment in regulatory drug trials.

## 1. Introduction

Mobility is a very broad term describing a person’s ability to change positions and move about in their home and community. Walking represents an important aspect of mobility that is commonly affected by ageing and chronic disease. Walking is a complex activity that requires intact musculoskeletal, neurological, and cardiorespiratory systems, as well as good sensory and neuromotor functions for effective performance [1]. Changes in any of these systems as a result of ageing and/or disease are reflected in walking performance. As such, walking speed is considered an additional vital sign of health [2] and represents an appropriate mobility measure for multiple populations. This is mirrored by strong evidence that mobility outcomes, such as walking speed and duration, predict mortality, falls, cognitive impairment, disability and other clinical outcomes [3,4,5,6,7]. As new therapies place more and more focus on multimorbid elders and on improving their quality of life and independence, the importance of assessing how a new medical product impacts mobility is growing exponentially. This was recently confirmed by the call for proposals “IMI2-2017-13-07—Linking digital assessment of mobility to clinical endpoints to support regulatory acceptance and clinical practice” that funded the Mobilise-D project [8]. Mobility is particularly important for diseases involving a significant burden of mobility disability, such as Parkinson’s Disease (PD) [9,10], Chronic Obstructive Pulmonary Disease (COPD) [11], Multiple Sclerosis (MS) [12], and the recovery after a fragility Proximal Femur Fracture (PFF) [13].

Despite this, inconsistent testing procedures and wide variations in the so-called "norms" have prevented mobility outcomes from being widely adopted. Consequently, the assessment of mobility is inconsistent both within and between diseases, precluding a harmonised approach to the measurement and understanding of mobility disability. Many clinical trials that support the marketing authorisation of a new medicinal product (hereinafter simply referred to as regulatory clinical trials) include some Patient-Reported Outcomes (PRO) or some simpler questionnaires aimed to evaluate the patient’s *mobility perception*. In PD studies, the second part of the Movement Disorders Society-sponsored new version of the Unified PD Rating Scale (MDS-UPDRS part II) is frequently considered the gold standard [14,15,16,17]. In COPD, the Saint-George Respiratory Questionnaire (SGRQ), which includes an activity domain assessing the effects of breathlessness on mobility and physical activity, is frequently used [18,19]. In MS, a widely used scale is the EDSS, in which the higher scores focus on walking disability [20]. Clinical trials of osteoporosis drugs have historically neglected the mobility aspect or any event after the hip fracture. However, in geriatrics and in general cost-benefit analyses, the use of a general quality of life questionnaire (EQ-5D), in which a component is focused on mobility, is widely accepted. 

Another approach to evaluate mobility in regulatory trials is the use of clinical tests to evaluate the patient’s *mobility capacity*. In PD, MDS-UPDRS part III is a clinician-assigned score based on capacity tests [14,15]. For COPD, the guidelines of the European Medicine Agency (EMA) recommend using cycle ergometry, or the 6-Minute Walking Test (6MWT) [21]. For MS, the timed 25-Foot Walk (T25-FW) component of the Multiple Sclerosis Functional Composite (MSFC) [22] is frequently used. For PFF, the Short Physical Performance Battery (SPPB) is frequently used [23,24]. 

Another dimension of mobility that is important is *mobility performance.* This can be defined as the volume and intensity of mobility that the patient performs during their daily life, as evaluated for extended periods in real-world conditions. For a long time, measuring this was technically impossible, but the introduction of wearable Inertial Measurement Units (IMU) has made this possible [8,9,25,26]. The Biomarkers, EndpointS and other Tools (BEST) glossary [27] defines a *biomarker* as “a defined characteristic that is measured as an indicator of normal biological processes, pathogenic processes, or responses to an exposure or intervention, including therapeutic interventions.” A *mobility outcome* is a biomarker that provides a quantification of each patient’s mobility. For example, according to this definition, the distance travelled during a 6MWT can be considered a mobility outcome. 

An emerging way to assess mobility performance is using Digital Mobility Outcomes (DMOs), which are mobility outcomes that are obtained by processing the recordings of wearable IMU, in order to extract specific features of the individual walking dynamics. Unfortunately, the first generation of these technologies was primarily designed for the consumer market as a motivational tool for young, healthy, fit and physically active adults [28,29,30,31]. When used as measurement tools, many of these sensors are very inaccurate [32,33], and even the most accurate become unreliable when used to monitor mobility performance in patients with some degree of mobility disability, particularly when walking at very slow speeds [34,35]. This is because the algorithms were developed using healthy and fit persons [25,33,36,37]. This has spurred intense research activity, which has produced a new generation of multisensor IMUs. Even more important is that this has led to a whole new class of analytical software that can more reliably detect the step events from the sensor’s raw signals, even in patients with severe mobility disability [38,39,40]. Despite this, to the authors’ knowledge, there is currently no DMO qualified for use as a mobility performance biomarker in regulatory drug trials by any regulator worldwide (with the only notable exception being a recent EMA qualification opinion on their use as secondary endpoint [41]). Before this can happen, it is necessary to demonstrate, for each specific Contexts of Use (CoU) within the regulatory process, that these DMOs are technically and clinically valid. In regulatory science, a CoU describes the appropriate use of the biomarker and how the qualified biomarker is applied in the regulatory review. 

So far, only a few attempts have been made to achieve regulatory qualification of DMOs, each with a fairly different approach. The PROactive consortium achieved a positive Qualification Opinion from EMA [42], where DMO recordings were combined with PRO data in a ‘PROactive Physical Activity in COPD (PPAC)’ tool that assessed patients’ experience of physical activity [43]. Of note, PPAC does not quantify mobility performance but patients’ perception of it. A second attempt was made by Trium Analysis Online GmbH, who submitted a letter of intent to the United States Food and Drug Administration (FDA) proposing to develop a digital health technology clinical outcome assessment (COA) to evaluate a change in continuous real-world walking speed in patients with Multiple Sclerosis [44]. Whereas the information provided at this stage was limited, the response from the FDA implied that the aim of this submission would be much more ambitious, e.g., to position DMOs as biomarkers on their own, which quantify a real-world mobility construct that could be considered an endpoint on its own. These early attempts confirm that there is no clarity within the human movement research community on how to best position the use of an IMU in regulatory trials of new drugs. Since this regulatory science debate is largely precompetitive, the Mobilise-D consortium felt it appropriate to report the details of the qualification plan that it recently developed for the benefit of the wider community.

The aim of this perspective paper is to describe the qualification plan that the Mobilise-D consortium has developed to this purpose—a plan that has received a positive qualification advice from the EMA. We present this plan using, as a guiding example, the qualification of the use of DMOs as monitoring biomarkers to evaluate the mobility performance of patients with a confirmed diagnosis of Parkinson’s Disease. Even if such qualification evaluation has not yet taken place, the authors consider it important to share this experience as widely as possible to enable other groups to pursue similar qualifications for other contexts of use.

## 2. Qualification Strategy

There are at least two strategies to pursue the qualification of DMOs. The first is to propose mobility performance as a clinical outcome in its own right and use a DMO as quantification of such a primary clinical endpoint, much like in a weight loss study where the measured change in weight at a given follow-up time with respect to a baseline is a quantification of the endpoint. This approach was used in the request for qualification DDT COA #000106 submitted to the United States Food and Drug Administration (FDA) [44]. However, in the review of the letter of intent, the FDA requested that, in addition to an extensive technical validation, the proponents should “ensure the endpoints represent outcomes that are meaningful to patients.” In our research community, it is considered a self-evident truth that mobility is something that, like being alive and free of disease, is something every human desires. However, this notion was challenged by the regulator, who required it to be explicitly demonstrated. This poses a challenge. For example, which features of mobility performance are more important for which patients? For example, is it more important to walker faster or farther? The key benefit for a 20-year-old athlete will be quite different from that for an 80-year-old retiree, etc. Answering such questions would require an extensive qualitative research programme. Another issue is that usually in a regulatory clinical trial a clinical endpoint is a direct and intended target outcome for the drug being tested. However, drugs usually affect mobility only indirectly.

The second strategy is more conservative, as it does not assume mobility as being a target outcome for the drug being tested. For diseases where the evaluation of mobility perception and/or mobility capacity are already accepted as elements of constructs used to monitor the state of the disease, adding quantification of mobility performance as a secondary monitoring biomarker or as a surrogate of an accepted clinical endpoint can be proposed. In this case, it is necessary to demonstrate, in addition to the technical validity, the clinical validity of the new biomarker by showing construct validity, predictive capacity and the ability to detect change. In the first stage, the validity of a specific DMO as a monitoring biomarker will be demonstrated for a single disease-specific CoU. In the second stage, this will be repeated for multiple diseases, along with exploring a single DMO which shows validity across multiple diseases. In the third stage, the ability of a DMO to serve as a clinical endpoint, and as surrogate of accepted clinical endpoints, is more difficult, expensive and time-consuming to observe than the DMO, can be explored. This strategy is the one that the Mobilise-D consortium has pursued in seeking the qualification of novel methodologies for the drug development of EMA.

## 3. Qualification Process

Mobilise-D is a consortium of 19 academic institutions and 14 companies formed to pursue the project “Connecting digital mobility assessment to clinical outcomes for regulatory and clinical endorsement” funded by the European Commission through the program H2020-EU.3.1.7.—Innovative Medicines Initiative 2 (IMI2). Within the consortium we formed a Qualification Task Force (QTF) that included industrial and academic expertise on regulatory science, human movement analysis and biomechanics, clinical epidemiology and statistics, health informatics, as well as clinical research expertise in all four diseases of interest.

**Figure 1:**
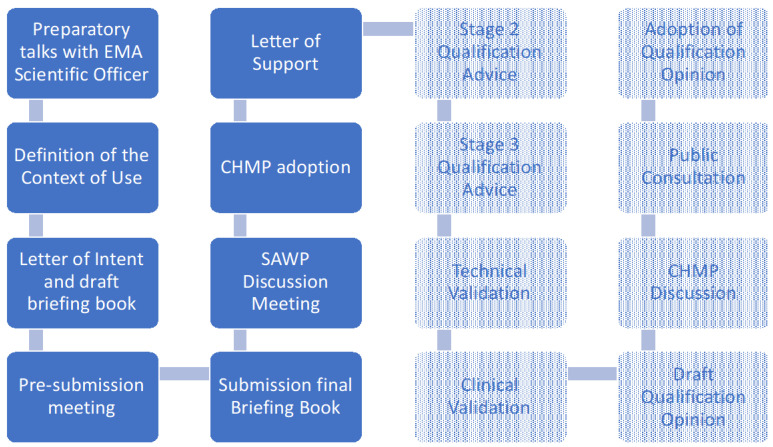
The complete workflow European Medicine Agency (EMA) qualification of novel methodologies for medicine development. The blue boxes are covered in this paper.

The complete process for the EMA qualification of novel methodologies for medicine development is depicted in Figure 1.

The QTF had preparatory talks with a scientific officer (EMA Human Medicines Research & Development Support Division) with specific expertise in digital health, and with the chair of EMA Innovation Task Force (ITF). During these conversations the qualification strategy described above was defined, based on three consecutive stages of advice. A key element of the qualification strategy was the definition of the Context of Use for which we requested the qualification of the new methodology. 

A first draft of the stage 1 briefing document was submitted together with a letter of intent, and a preparatory meeting between the QTF and the EMA scientific advice office took place. Based on the comments we received, the final briefing document was submitted for the attention of the Scientific Advice Working Party (SAWP). Because of the complexity of the technical validation protocol presented, the EMA qualification team included two experts of human movement analysis and biomechanics.

At its January 2020 meeting, the SAWP adopted a list of issues to be addressed by the Applicant in writing and during the discussion meeting. The QTF submitted the written responses and addressed the list of questions during a discussion with the EMA qualification team in the framework of the SAWP meeting. The draft report prepared by the qualification team coordinator in consultation with the qualification team members and enriched by the interactions with the applicant was reviewed in the plenary session of the SAWP. During its meeting held on March 2020, the CHMP adopted the advice to be given to the Applicant.

The detailed Validation Plan described below is based on the stage 1 briefing document that was submitted to the SAWP, modified where necessary in order to reflect the qualification advice EMA provided. 

In a separate section, we also provided a description of how we plan to generalise the protocol to include multiple diseases, the exploration of a single multidisease DMO and the validation of the use of DMOs as surrogate biomarkers. These additional contexts of use are in the scope of stage 2 and stage 3 submissions, which are currently ongoing. Thus, for those contexts of use, no EMA advice has been provided yet. Once the Qualification Advice process is completed, we will start the technical and clinical validation activities. We expect those to take at least 30 months. The results will be submitted to the EMA for CHMP discussion, and a draft of the Qualification Opinion will be published for public consultation, which will last 60 days. If no substantial criticism is raised, the new methodology will then receive a positive Qualification Opinion. After that, any proponent will be allowed to use this methodology in any future regulatory clinical trial where the context of use is relevant. 

## 4. Results

### 4.1. Context of Use

We intend to pursue regulatory qualification for a new methodology developed by the Mobilise-D consortium to quantify mobility performance over a week in real world settings, using wearable sensors. We intend to use this measurement as an additional (i.e., secondary) monitoring biomarker to account for mobility performance in assessing the efficacy of new treatments for Parkinson’s Disease (PD) patients, complementing those already in use, which account only for patient’s perception of mobility and mobility capacity. 

### 4.2. Technical Validation

**Figure 2:**
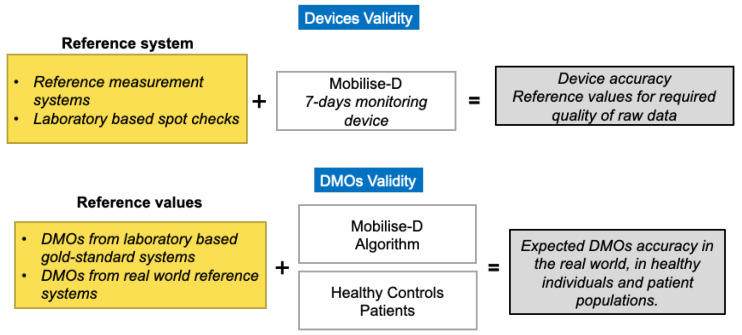
Mobilise-D Approach to test devices and DMOs validity.

The technical validation will be conducted on both healthy and diseased patients (in this case PD patients), using an approach based on a clear separation between the error associated to the measurement device from the error associated to the algorithms for the estimate of the DMOs (Figure 2). The results from the disease group will be used to quantify the accuracy of the system, whereas those from the healthy group will be used only as a comparison. 

To this purpose, we first established a procedure for metrological characterisation and quality check to characterise the adopted IMUs. This procedure will include a series of spot-checks based on the IEEE 2700-2017 Standard for Sensor Performance Parameter Definitions [45], which will allow the metrological performance of the accelerometers, gyroscopes and magnetometers included in the IMU to be defined. The focus will be on the noise first- (mean value) and second-order statistics (variance and Root Mean Square (RMS)), and the Root Allan variance parameters of noise [46,47], both under static and dynamic conditions. 

We then identified a multistage validation procedure to establish the precision and accuracy of the algorithms implemented to estimate the DMOs of interest in both healthy and pathological walking gait, as associated with the presence of PD. This will include a series of observations, ranging from testing healthy individuals under optimal (laboratory-based, prescribed simple walking tasks) and suboptimal (laboratory-based, unprescribed complex motor tasks) conditions to testing patients with pathological gait as in their own daily life scenarios (unsupervised, home, work or other habitual settings). For each of these contexts, we identified the optimal reference measurement tool: A stereophotogrammetric system (in conjunction with a series of ad hoc spot-checks to characterise its performances [48,49]) will be used as the laboratory-based gold standard under optimal and suboptimal conditions. The accuracy of state-of-the-art stereophotogrammetric systems is usually <1 mm when more than six cameras are used and the whole volume of capture is properly calibrated before beginning of the experiments [48,49]. The same system will also be used to establish the metrological properties and the accuracy of the DMOs estimated with a wearable multisensor system (INertial module with DIstance Sensors and Pressure insoles, INDIP [50,51,52]), which will then be used as a reference for the daily life scenarios. The INDIP system integrates inertial modules, distance sensors and pressure insoles. The inertial module includes an ultra-low-power microcontroller unit (MCU), a MIMU (→ triaxial accelerometer, gyroscope and magnetometer), flash storage and wireless connectivity (Bluetooth LE). Each distance sensor integrates an infrared time-of-flight distance sensor that can be connected to the inertial module by cable. The pressure insole consists of 16 resistive passive sensing elements. 

These series of observations, during which the patients will be instrumented with the IMU to be tested, will evaluate how the accuracy and precision of the IMU for each DMO changes when moving from the laboratory to the real world and from healthy to pathological gait. The technical validation will be conducted in five different laboratories, located in three different European countries.

### 4.3. Clinical Validation

For each DMO, the qualification protocol requires assessing construct validity, predictive capacity, and ability to detect change. We use the term construct to indicate a proposed attribute of a person that often cannot be measured directly but can be expressed using several indicators or manifest variables. In our case, the construct to be tested is the concept of mobility performance, expressed by the various DMOs. 

In order to be valid, a construct must show convergent validity, separation between known groups, and discriminant validity. In this case, convergent validity is tested by correlating each DMO with the MDS-UPDRS II and MDS-UPDRS III with a priori strength of correlation determined from the literature. Separation between known groups is determined by the Hoehn & Yahr (H&Y) scale [53], which indicates increasing stages of motor disease severity that result in a reduction of the construct mobility performance [15]. If this is the case, each DMO should have a different distribution among different H&Y stages. To demonstrate discriminant validity, we will confirm that each DMO does not correlate with constructs that are unrelated to mobility performance. For example, PD patients may experience changes of tremor occurrence and frequency during the course of the disease, but they do not interfere with mobility, so the DMO should not correlate with changes in tremor occurrence and frequency.

The predictive capacity of the construct will be evaluated by testing the correlation between the baseline levels of each DMO (and their changes over time) and changes in the MDS-UPDRS total score, as well as its sub-scores.

The ability to detect change is tested by evaluating the ability of each DMO to detect true changes over time, which, in this case, will be any reported change of medication that has occurred. We will also quantify the minimal important difference (MID), i.e., the smallest change in each DMO that is positively associated with the perception of the patient or the clinician of an improvement or a worsening of the condition. We will determine the MID using the UPDRS-II (which is patient-reported) and UPDRS-III (which is clinician-reported) as anchors. Last, the responsiveness of the DMO will be tested in relation to interventions that are known to be effective, and the effect size between them will be calculated using Cliff’s delta [54].

The clinical validation will be based on a multicentre observational study. PD patients will be recruited in five centres across four European countries to represent diverse health care systems, geographical areas and different socioeconomic regions. We aim to include patients from different strata of gait speed between 0.4–1.2 m/s. The participants will be followed for 24 months and invited for five visits, including one home-based assessment to understand contextual factors, such as the build environment and its accessibility, usability and neighbourhood walkability. As the central digital component, all participants will wear a body-worn sensor for a full 7 days. To have sufficient power to analyse the accuracy of different DMOs on monitoring, evaluation, stratification, prediction and prognostic modelling, 600 participants will be recruited.

EMA qualification advice confirmed that the observational clinical validation study was adequate to assess construct validity and predictive capacity. Regarding the ability to detect change, the observational study was considered adequate to validate the longitudinal validity and to quantify the MID. However, as expected, some reservations were expressed around the ability to assess responsiveness with an observational design where the allocation of treatment is not random but determined by changes in usual care for each patient if and when their condition requires it. We intend to add the Mobilise-D protocol to quantify DMOs in interventional randomised clinical trials and use their results to evaluate the responsiveness in a randomly assigned, controlled way.

### 4.4. Secure Data Management

To enable the Technical and Clinical validation phases described above, we have to implement a fit-for-purpose process that facilitates a standardised approach to capture, transfer, ingestion and integration, storage, and analysis of the data that is required for the qualification process. Furthermore, we need to adhere to relevant legal frameworks and implement appropriate data access and governance models. This is a complex process, as the Mobilise-D programme of research involves large multimodal datasets that are being sourced from over 2500 patients, over as long as a 2-year period, in multiple clinics and laboratories across many countries. 

A critical guiding principle for all aspects of the Mobilise-D pathway toward qualification is that we adhere to the highest standards of data integrity in all of our activities. In this regard, the Mobilise-D consortium has adopted the ALCOA+ data integrity principles [55]. Adherence to ALCOA+ requires that all data records are attributable, contemporaneous, original, accurate, complete, consistent, available and enduring. An example of the practical impact of these principles is the requirement for all original source data to be integrated and maintained indefinitely on a secure platform in a manner that preserves the data and privacy rights of the study participants. We also need to implement a rigorous quality control process to ensure that data records are accurate and complete. 

In addressing data privacy and security requirements, we are adhering to the GDPR and Directive 2006/24/EC [56,57] and have implemented a series of measures to protect the privacy and data rights of study participants throughout the process. All data will be integrated, managed and stored using unique identifier codes. All programme algorithms and data will be stored on e-Science Central, which is a scalable cloud-based platform that supports secure storage, analysis and sharing of multimodal data [58]. In Mobilise-D e-Science Central will be implemented on Amazon Web Services. All data will be encrypted in transit and at rest. Flat files will be stored in S3 buckets which are encrypted using AES-256 encryption. Amazon relational databases will also be encrypted using AES-256. A data governance and access control policy will be implemented to ensure appropriate management and access throughout the Mobilise-D programme. 

## 5. Discussion

The aim of this paper was to describe the plan for regulatory qualification of the use of DMOs as monitoring biomarkers to account for mobility performance in Parkinson’s Disease (PD) patients, complementing those already in use, which account only for patient’s perception of mobility and mobility capacity. Such plan has received a positive qualification advice from the EMA.

The plan involves an extensive technical validation using a classic metrology approach and a clinical validation that involves the evaluation of construct validity, predictive capacity, and ability to detect change. Upon request of qualification advice to EMA, this plan received largely positive suggestions.

A general limitation of the use of DMO in relation to Parkinson’s Disease is its poor specificity. Whereas most PD patients eventually develop mobility disabilities, not all patients with mobility disabilities are affected by PD. Thus, while DMOs are well suited to monitor the progression of mobility disability in patients with a confirmed diagnosis of Parkinson’s Disease, DMOs alone cannot be used for PD diagnostics.

This approach to regulatory approval has several limitations. The most important is that the context of use for which we obtained an EMA positive qualification advice is limited to Parkinson’s Disease. The authors are convinced that different DMOs are likely to be effective monitoring biomarkers for several diseases involving mobility disability. Furthermore, there is a concrete possibility that a single DMO might be an effective monitoring biomarker also across multiple diseases. This would provide a disease-independent monitoring biomarker for mobility performance that could be relatively easy to extend to other diseases not considered in the Mobilise-D project. Indeed, that is the hope upon which the project was funded—that the intrinsic nature of the relationship between mobility and health outcomes persists across multiple diseases, and that this can be measured with a single DMO, although perhaps with different cutoffs for elevated risk. 

Another limitation is that, according to various authors, DMOs could be used as surrogate biomarkers for important clinical endpoints, such as admission to care-homes for PFF patients and falls for MS and PD patients. Whereas, in some cases, it is possible that the predictive power of a DMO for such endpoints is high enough to validate its use as surrogate biomarker, in others, it is possible that such predictive power can only be achieved with the DMO in composite with other biomarkers. 

The third limitation is that the qualification plan proposed here does not consider the possibility of pursuing the qualification of mobility performance, measured in the real world over a few days, as a clinical endpoint in itself. Although it may seem obvious that an improvement of average mobility reflects a desired effect of a new drug, and a reduction of mobility reflects an unwanted, or even adverse effect, regulatory bodies do require evidence to support each statement. To pursue such a qualification, it would be necessary to demonstrate convincingly that patients consider any increase of mobility as a desired effect, and any loss of mobility as an undesired effect. 

Some of these limitations will be addressed in the follow-up work that the Mobilise-D consortium is undertaking, according to the staged qualification approach described in the “Qualification Process” section above. Such a stepwise approach was recently praised in a paper co-authored by EMA scientific officers [59]. In a second request for EMA qualification advice, which is currently in preparation, we plan to use the same approach described here for the PD to seek qualification for the use of DMOs as monitoring biomarkers in MS, COPD, and PFF. In addition, we will seek EMA scientific advice on the possible use of a single DMO as a monitoring biomarker of mobility performance across all four diseases, using the Late Life Functional Disability Index (LLFDI) as an overarching instrument to assess disability and mobility, in particular, to allow a disease-independent validation. Last, we plan to explore the possibility of using DMOs as surrogate biomarkers predictive of disease-specific clinical endpoints.

In conclusion, we proposed an incremental approach to regulatory qualification that could help other areas. We hope that this first EMA qualification advice, in combination with the recently published EMA Q&A on the qualification of digital technologies [60], can finally broaden the use of IMUs in drug trials.
